# Ophthalmology Research Trends in Saudi Arabia: A Four-Decade Bibliometric Analysis

**DOI:** 10.7759/cureus.11465

**Published:** 2020-11-13

**Authors:** Saeed Alshahrani, Adi Mohammed Al Owaifeer

**Affiliations:** 1 Otolaryngology, College of Medicine, King Saud Bin Abdulaziz University for Health Sciences, Riyadh, SAU; 2 Ophthalmology, College of Medicine, King Faisal University, Al-Ahsa, SAU; 3 Glaucoma Division, King Khaled Eye Specialist Hospital, Riyadh, SAU

**Keywords:** bibliometric analysis, ophthalmology, bibliometrics, research publications

## Abstract

Background

Clinical research output by a country could reflect its advancement in medical education and quality of patient care. This study aims to assess the contribution of Saudi research in the field of ophthalmology.

Methods

This retrospective cross-sectional bibliometric analysis was performed in March 2020 to assess the contribution of Saudi research in the field of ophthalmology. Web of science (WoS) advanced search engine was used. All Saudi-affiliated publications from 1980 till 2019 were included. A qualitative assessment included the total number of publications, citations, citing articles, journal titles, and affiliated institutions. Quality of publications was evaluated by H-index. A comparative quantitative analysis of Arabian and some selected countries worldwide was performed. Publication productivity was adjusted to each country by population size.

Results

In the period of 1980-2019, Saudi Arabia published 2178 articles in ophthalmology. The number of publications (Spearman's correlation Coefficient = 0.94; P < .001) and sum of citations (Spearman's correlation Coefficient = 0.99; P < .001) increased significantly over the past 40 years. The highest notable increase was detected in the period for 2010-2019. H-index was 58 with an average citation of 10.79 per article. The sum of citations was 23,507 while 18,460 articles were cited by Saudi publications. When compared to other countries, Saudi Arabia ranks the highest among Arab countries and the second most productive in Asia following Japan.

Conclusion

Despite the continuous improvement in the trends of ophthalmology research in Saudi Arabia, further effort is needed to strengthen the publications output and achieve a considerable international status.

## Introduction

Located in Western Asia, Saudi Arabia is the second-largest Arabian country. According to the General Authority for Statistics (2020), it has a population of 34.81 million, making it the 41st most populous country in the world. Saudi Arabia is greatly blessed with oil and natural gas, resulting in an extraordinary economic evolution that has contributed to notable improvements in the quality of education, healthcare, and many other living standards [1].

In the new era of technology and digital libraries, bibliographical tools have become more advanced, accessible, and easy to use. Scientometrics, which originated in the early 19th century and expanded slowly over decades, experienced a rapid shift at the beginning of the 21th century when the internet became accessible for all [2]. Moreover, the utility of bibliometrics in the medical literature increased 57-fold over the past 20 years [3]. Nowadays, the most commonly used databases to manage and report bibliometric research projects include the Web of Science (WoS) (1966-present), Scopus (2004-present), and Google Scholar (2006-present).

In 2003, one of the first global publications to apply bibliometric principles in ophthalmology was conducted in Japan, covering the 15 years from 1988 to 2002 [4]. This study revealed that 49.5% of ophthalmology research came from North America, while 31.3% came from Western Europe, 15.1% from Asia, 2.2% from the Middle East, 0.85% from South America, 0.53% from Eastern Europe, and 0.47% from Africa [4]. In 2012, Sweileh et al. reviewed the status of Arabian publications in ophthalmology, an analysis that was limited to original research papers and review articles only [5]. Their results revealed that the total number of articles authored or co-authored by Arab researchers was 2,083. Furthermore, Arabian research papers had an H-index of 51 and an average citations per document of 10.13 [5]. In addition, this study revealed that 39.75% of Arabian publications came from Saudi Arabia. However, a qualitative assessment of Saudi contributions to ophthalmology was not conducted.

Despite the notable advancement of ophthalmology in Saudi Arabia over the past few decades, the peer-reviewed literature lacks a detailed description of the quality and quantity of Saudi ophthalmology research from a bibliometric perspective. Therefore, the aim of the current study was to explore the trend of ophthalmology publications in Saudi Arabia - compared to regional and global contributions - over the past 40 years.

## Materials and methods

Study setting & design

The WoS advanced search engine was our tool of choice to conduct the bibliometric search since it provides a standard dataset to analyse and track bibliographical parameters, including author names, keywords, affiliation, country, journal title, number of citations, and broad subject categories. Moreover, WoS contains 1.6 billion cited references from 1900 to the present day [6]. Data used in the current bibliometric analysis were retrieved from the WoS core collection during the period 1980 to December 2019. Data recruitment was performed in March 2020. As the data were downloaded from available published research, no ethical approval was required.

Search strategy

Using the advanced search engine, the Web of Science category tag was defined as “ophthalmology”, and was then combined with each country and evaluated separately. The status of Arabian publications was identified using country tags, as follows: “Saudi Arabia” OR “Egypt” OR “Tunisia” OR “Lebanon” OR “Morocco” OR “United Emirates” OR “Oman” OR “Jordan” OR “Kuwait” OR “Qatar” OR “Algeria” OR “Syria” OR “Sudan” OR “Iraq” OR “Bahrain” OR “Palestine” OR “Yemen” OR “Libya” OR “Somalia” OR “Mauritania” OR “Comoros” OR “Djibouti”. Other countries included were “United States”, “England”, “Switzerland”, “Australia”, “Canada”, “Germany”, “France”, “Japan”, “Italy”, “Iran”, “Brazil”, “China”, “Russia”, “India”, and “Turkey”. The validity of the search strategy was tested by examining the retrieved parameters manually.

Data analysis

Data were imported to Microsoft Excel (Microsoft® Corp., Redmond, WA) for analysis. Quantitative variables retrieved included the total number of publications, citations, citing articles, journal titles, and organization-enhanced publications. The expected number of publications in 2030 was estimated by applying an equation that correlates our ascending variables. Using Excel, equation reliability was visualized through a polynomial trendline with an R2 of 0.9 for the expected number of citations and a linear trendline with an R2 of 0.75 for the expected number of articles. SPSS (IBM Corp., Armonk, NY) was used to measure Spearman’s correlation coefficient, and a p-value of less than 0.05 was considered significant. For qualitative analysis, we applied the Hirsch equation (H-index), which is the measurement most widely used to describe simultaneously the quality and quantity of a publication group. The H-index was evaluated using the Citation Report in the Web of Knowledge. We compared the number of published ophthalmology documents among all Arabian countries in addition to several highly-cited countries worldwide. We also analysed publication productivity by adjusting for population status in each country. The number of publications was adjusted to the population of each country using data extracted from United Nations Population Division Estimates (2019) [7].

## Results

Quantitative bibliometrics

**Figure 1:**
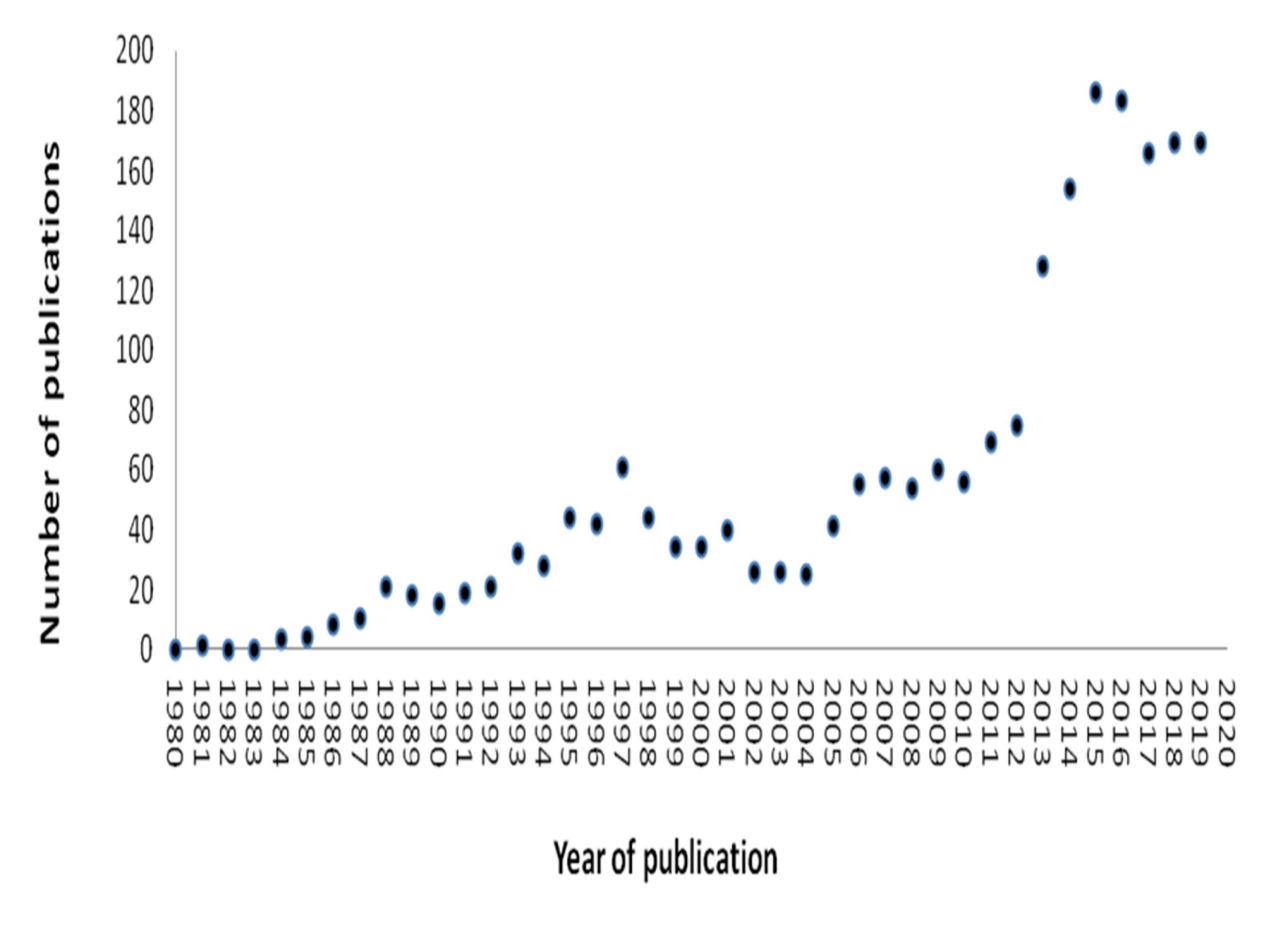
Total number of publications per year from 1980 to 2019. R2 = 0.75, Spearman's correlation Coefficient = 0.94; p-value < 0.001.

During the period 1980 to 2019, Saudi-affiliated ophthalmologists published a total of 2,178 articles. The annual number of published articles was around 20 during the 1980s, and then started to increase gradually over the following two decades, reaching slightly over 60 articles a year in 2010. The annual number of publications then soared during the last decade of the studied timeframe, reaching 180 articles in 2019. Using Spearman's correlation coefficient, we note a positive trend in the number of new articles per year, and this trend reached statistical significance (P < .001) (Figure 1). Based on the linear equation derived from the number of publications within the period 2010-2019, we expect the number of new publications by 2030 to be nearly 367 articles per year (R2 = 0.75, Spearman's correlation coefficient = 0.94).

**Figure 2:**
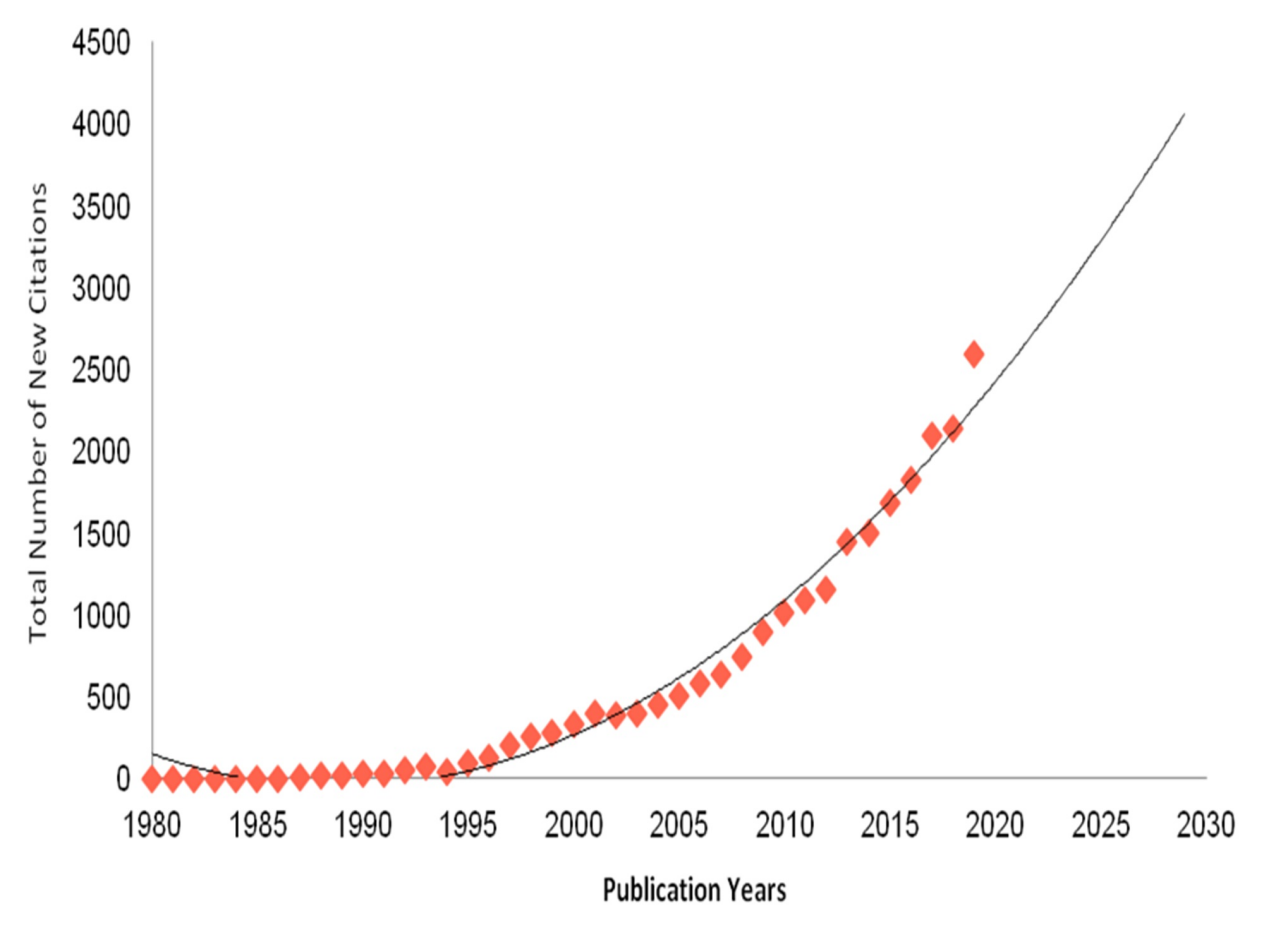
Total number of citations per year from 1980 to 2019. R2 = 0.98, Spearman's correlation Coefficient = 0.99; p-value < 0.001.

Over the last 40 years, the number of yearly cited articles grew steadily until 1995, at which point it started to accelerate quickly, representing a polynomial non-linear trendline (R2 = 0.98, Spearman’s correlation coefficient = 0.99) (Figure 2). This growth pattern is statistically significant (P < 0.001). Moreover, the estimated number of citations per year is expected to double by 2030. The H-index of the studied articles was 58, with an average of 10.79 citations per article. The total number of cited Saudi articles was 23,507, 21,904 of which lacked self citations. On the other hand, Saudi ophthalmologists cited 18,460 articles, with 17,734 of which lacked self citations.

Articles from Saudi-affiliated researchers were published in more than 100 journals in the field of ophthalmology. The journal with the largest number of citations is Investigative Ophthalmology & Visual Science (307), followed by the Saudi Journal of Ophthalmology (127), and the British Journal of Ophthalmology (116). Further details are shown in Table 1.

**Table 1:** Top 10 publishing journals for Saudi-affiliated ophthalmologists from 1980 to 2019.

Rank	Journal name	Number of publications
1st	Investigative Ophthalmology & Visual Science	307
2nd	Saudi Journal of Ophthalmology	127
3rd	British Journal of Ophthalmology	116
4rth	Ophthalmology	104
5th	American Association for Pediatric Ophthalmology and Strabismus	92
6th	American Journal of Ophthalmology	84
7th	Middle East African Journal of Ophthalmology	84
8th	Ophthalmic Genetics	79
9th	Acta Ophthalmologica	60
10th	Cornea	60

Status of ophthalmology research in the Arab world

**Figure 3:**
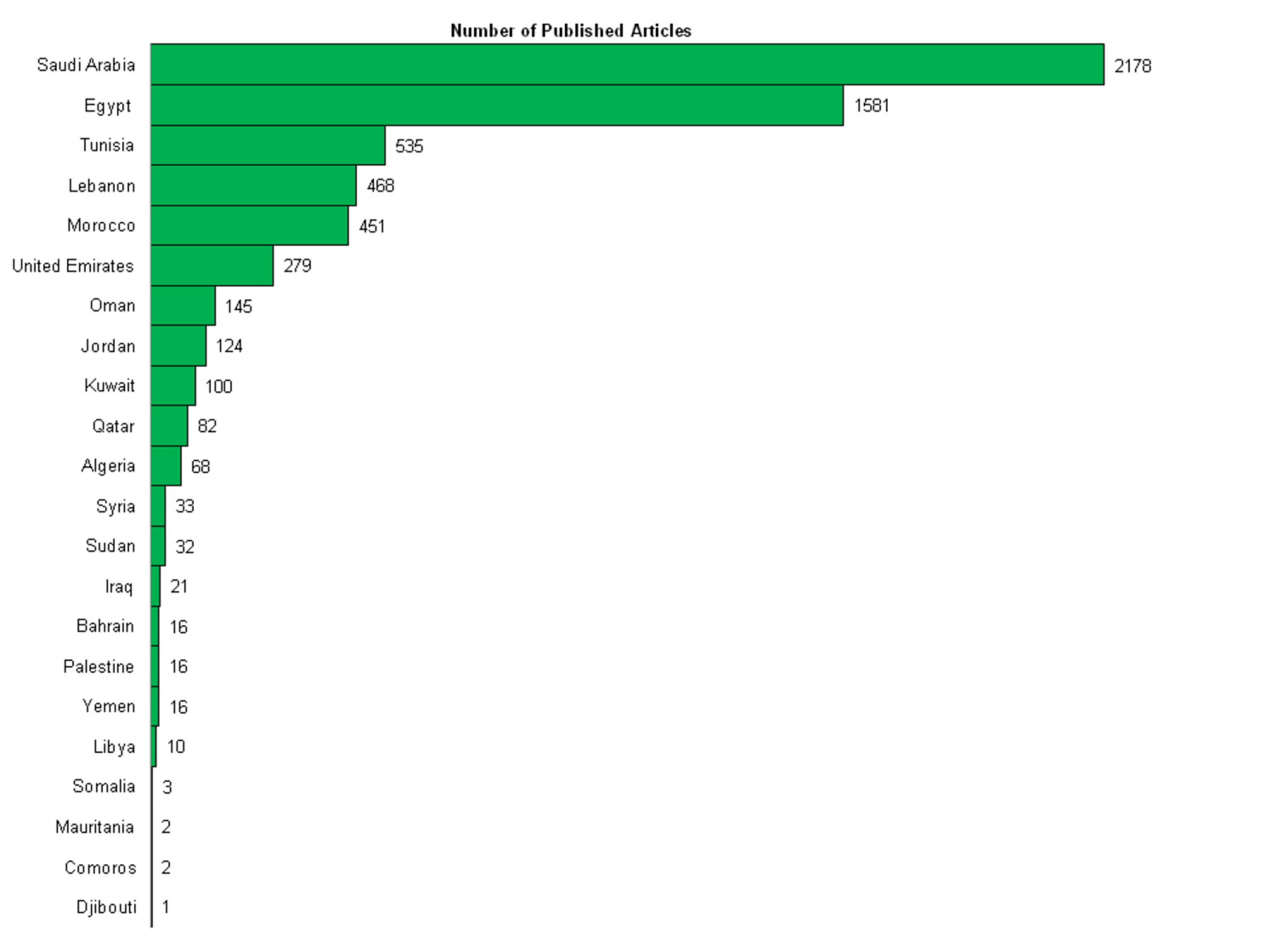
Number of ophthalmology publications in the Arab world from 1980 to 2019.

Figure 3 shows that the total number of ophthalmology publications in the Arab world during the studied time period was 6,163 articles. The majority (35%) of the Arabian research output came from Saudi Arabia. Egypt had the second largest number of publications among Arab countries with 1,581 articles (25.7%), followed by Tunisia with 535 (8.7%). The most productive institutions in the Arab world were King Saud University (1,585 articles, or 25.7%) and King Khaled Specialized Eye Hospital (1,096 articles, or 17.8%). Four of the top 10 most active centres are located in Saudi Arabia. Further details about the top 10 active research institutions within the Arab world are shown in Table 2.

**Table 2:** Top 10 most active Arabian institutions in ophthalmology research from 1980 to 2019.

Rank	Institution	Number of publications n = 6163 (100%)	Country
1st	King Saud University	1585 (25.7%)	Saudi Arabia
2nd	King Khalid Eye Specialist Hospital	1096 (17.8%)	Saudi Arabia
3rd	King Khalid University Hospital	992 (16%)	Saudi Arabia
4rth	Cairo University	368 (6%)	Egypt
5th	American University of Beirut	368 (6%)	Lebanon
6th	University of Tunis El Manar	293 (4.8%)	Tunisia
7th	General Organization for Teaching Hospitals & Institutes	238 (3.9%)	Egypt
8th	Research Institute of Ophthalmology	233 (3.8%)	Egypt
9th	Alexandria University	228 (3.7%)	Egypt
10th	King Abdulaziz University	217 (3.5%)	Saudi Arabia

Global experience in ophthalmology research

**Figure 4:**
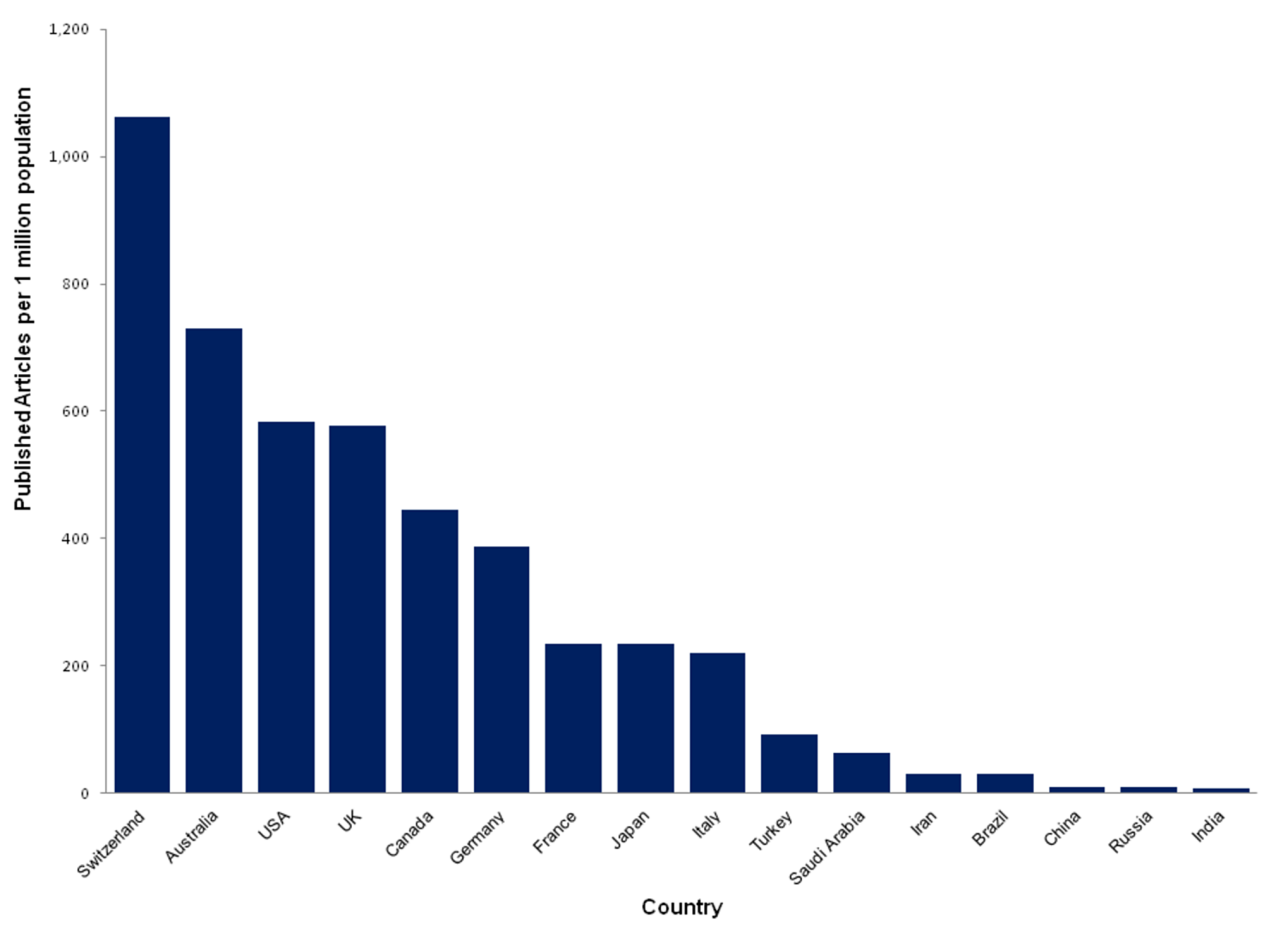
Productivity of ophthalmology publications in selected countries worldwide from 1980 to 2019.

A total of 530,488 articles in the field of ophthalmology were published worldwide from 1900 to December 2019. The USA has the largest number of publications (191,755), which constitutes around 36% of the global published output, followed by the United Kingdom (38,938), Germany (32,314), Japan (29,677), Australia (18,382), Canada (16,658), France (15,335), and China (15,291). Figure 4 selectively compares the number of ophthalmology publications per million population between different countries. Switzerland ranks the highest, followed by Australia and the USA. Saudi Arabia ranks the second most productive country in Asia (following Japan) and the second most productive in the Middle East (following Turkey). Furthermore, according to Scimago Journal & Country Rank (SJR), Saudi Arabia ranks 26th worldwide in the number of published ophthalmology documents.

## Discussion

The application of scientometrics within the field of ophthalmology facilitates exploring the productivity of local medical institutions, thus helping to properly allocate future research funding. Moreover, discussing the productivity of Saudi research should positively motivate Saudi researchers, ophthalmologists, and institutions for further advancement. As a result of improvements in health informatics, scientometrics is now capable of examining the influence of publications through citation reports, knowledge mapping techniques, and other quantitative bibliometrics parameters [2,3].

This study is one of the first to utilize scientometrics to specifically describe the status of Saudi ophthalmology research. In a study conducted to analyse the output of Arab researchers in ophthalmology, Sweileh et al. previously reported the number of Saudi publications in ophthalmology (828 articles); however, their study did not provide an in-depth analysis of Saudi Arabia’s research output [5]. The current study was conducted because we believe that a comprehensive evaluation of H-index, number of citations, citations per article, with a particular focus on the growth behaviour of Saudi publications, is required. Our bibliometric analysis showed that the quantity and quality of ophthalmology research in Saudi Arabia escalated significantly over the last four decades. Saudi Arabia had by far the highest productivity in ophthalmology research among Arabian nations, and ranks favourably in the Eastern world.

According to our analysis, more than 20 Saudi institutions and universities were among the most active 100 Arabian organizations in the field of ophthalmology. King Saud University was the most active Arabian academic institution in ophthalmology publications. King Abdulaziz University was within the top 10 Arabian institutions and was also the second most active academic institution in Saudi Arabia. This evolution in ophthalmology research reflects how medical organizations and schools have enhanced the development of ideal productive research environments. In 2018, the government of Saudi Arabia allocated one-fifth of its total budget - an estimated US$54.8 billion - to the Ministry of Education. This budget may translate in the near future into a larger number of publications from academic institutions.

Launched in 2010, the Saudi Digital Library (SDL) is an online academic portal that contains more than 200 million scientific materials, including digital books, journals, periodicals, audio-visual items, and databases [8]. Recently, the Ministry of Education provided SDL access to the students and faculty members of all Saudi universities, a substantial step that will aid in advancing education and publications processes. Since all Saudi academic users can now access SDL, the use of bibliometric analysis to explore productivity of publications per field is expected to increase in the setting of easily accessible search engines such as PubMed, WoS, Scopus, Google Scholar, and many others.

In addition to academic institutions, hospital-based research centres supported by governmental agencies have shown great achievement in terms of productivity in ophthalmology research. An excellent example is that of King Khaled Specialized Eye Hospital (KKESH), in which a dedicated budget is maintained to support research-related activities. KKESH was established in 1983 and remains one of the finest tertiary hospitals providing ophthalmic care worldwide [9]. Additionally, the Saudi Ophthalmology Society (SOS), which was established in 1985, notably contributed research improvement by arranging local meetings and granting an annual award for the best research project [10]. In 1986, SOS launched the Saudi Journal of Ophthalmology, which is, along with the Saudi Medical Journal (1979) and the Annals of Saudi Medicine (1985), one of the oldest and most venerable journals in Saudi Arabia [10,11].

One explanation for the marked increase in Saudi-affiliated publications over the last 10 years (Figure 1) is that the number of practicing ophthalmologists has increased gradually over the last decade. In 1987, a total of 163 physicians practised ophthalmology in Saudi Arabia, for an ophthalmologist to population ratio of 1:55,000 [10]. In 2014, the number of ophthalmologists was estimated to be 407, for an ophthalmologist to population ratio of 1:43,000. The number of ophthalmologists is expected to reach 1,100 by 2020 [12]. In addition, the Saudi Commission for Health Specialties (SCFHS) recently implemented research project points as a requirement for applying to an ophthalmology residency position. Furthermore, research has been included as part of the curriculum for both residency and fellowship training programs [13].

Although the status of ophthalmology research in Saudi Arabia seems to be satisfactory, Saudi ophthalmologists must strive to improve the nation’s global research ranking and to attain optimal ophthalmic care to meet the expectations outlined in the historic Saudi Vision 2030. Several strategies could further develop current ophthalmology research practice. One suggestion is that academic institutions should prioritize the allocation of research funds based on a periodic centre-level bibliometrics analysis. Additionally, since a significant proportion of the medical research practice in Saudi Arabia results from extremely time- and resource-consuming individualized efforts, a devoted research department in each institution, comprising staff trained in conducting research, would add great value and help to overcome the many obstacles in research practice.

Our study has two limitations. First, due to technical issues related to discrepancies in the spelling or formatting of authors’ names, we were not able to gain an accurate estimation of the most active researchers in ophthalmology. Second, our analysis was based on data retrieved from only one database (i.e. WoS). Nevertheless, we believe that this is the best approach to fulfil our objectives since the application of more than one bibliographical database will yield significantly different results. Moreover, WoS remains the gold standard to conduct bibliometrics studies; it allows researchers to retrieve citation reports since 1990 without language restrictions, and these reports are clustered by individual, institution, country, and field.

## Conclusions

Ophthalmology publications in Saudi Arabia witnessed a positive trend within the studied time frame, with a significant increase in the number of published reports over the past decade. King Khaled Specialized Eye Hospital and King Saud University remained the most active institutions publishing ophthalmology research in the Arab world, with the latter being more active recently. Saudi Journal of Ophthalmology ranked second most active journal in publishing Saudi-affiliated articles following Investigative Ophthalmology & Visual Science.

Although the impact of Saudi publications is relatively low compared to global contributions, Saudi Arabia remains the leading Arabian country in ophthalmology research. The results of this analysis could act as a benchmark for future studies, help in resource allocation within the kingdom of Saudi Arabia, and promote further advancement in ophthalmic research.
